# ﻿*Hymenaspleniumtholiformis* (Aspleniaceae), a new fern species from southeastern Xizang, China based on morphological and molecular evidence

**DOI:** 10.3897/phytokeys.204.85746

**Published:** 2022-08-05

**Authors:** Yong-Lin Qiu, Ke-Wang Xu, Wen-Bin Ju, Wang-Lin Zhao, Liang Zhang

**Affiliations:** 1 School of Ecology and Environmental Science, Yunnan University, Kunming, 650091, China Yunnan University Kunming China; 2 Co-Innovation Center for Sustainable Forestry in Southern China & Key Laboratory of National Forestry and Grassland Administration on Subtropical Forest Biodiversity Conservation, College of Biology and the Environment, Nanjing Forestry University, Nanjing, 210037, China Nanjing Forestry University Nanjing China; 3 Key Laboratory of Mountain Ecological Restoration and Bioresource Utilization & Ecological Restoration Biodiversity Conservation, Chengdu Institute of Biology, Chinese Academy of Sciences, Chengdu, 610041, China Chengdu Institute of Biology, Chinese Academy of Sciences Chengdu China; 4 College of Science, Tibet University, Lhasa, 850000, China Tibet University Lhasa China; 5 Motuo Observation and Research Center for Earth Landscape and Earth System, Chinese Academy of Sciences, Linzhi 860712, China Chinese Academy of Sciences Linzhi China; 6 CAS Key Laboratory for Plant Diversity and Biogeography of East Asia, Kunming Institute of Botany, Chinese Academy of Sciences(CAS), Kunming, 650201, China Kunming Institute of Botany, Chinese Academy of Sciences Kunming China

**Keywords:** *Hymenasplenium*, *H.excisum* subclade, Medog, pinna morphology

## Abstract

A new species of Aspleniaceae, *Hymenaspleniumtholiformis* sp. nov., is described from Medog County in southeastern Xizang, China. The new species is morphologically similar to *H.apogamum* and *H.szechuanense*, but the former has ascending pinnae, pinna apex obtuse to rounded, pinna-marginal teeth entire, and veins terminating just below marginal teeth. Phylogenetic analysis based on five plastid markers confirmed that this new species represents a diverging lineage in the *H.excisum* subclade of *Hymenasplenium*.

## ﻿Introduction

*Hymenasplenium* Hayata is one of two genera in the species-rich fern family Aspleniaceae, comprising more than 60 species worldwide (Murakami and Moran 1993; Murakami 1995; PPG I 2016; Schneider et al. 2017; Xu et al. 2018b). The genus is characterized by the stelar structure of the long-creeping rhizome, dorsiventrally symmetrical steles, swollen petiole bases, and unique rachis-costae structure (Murakami 1995; Xu et al. 2018b). Extensive cryptic speciation in *Hymenasplenium* has been demonstrated based on recent studies (Chang et al. 2018; Xu et al. 2018b), which resulted in the discovery of a large number of new species (Chang et al. 2018; Xu et al. 2018a, 2019a, 2019b).

Located in southeastern Xizang, Medog County is one of the biodiversity hotspots in China, which has rich plant diversity in the Eastern Himalaya (Sun and Zhou 1996). Recently, new taxa of seed plants have continuously added to the flora of this region (Li et al. 2018; Li et al. 2021; Tian et al. 2021; Ya et al. 2021; Zhou et al. 2021). Previous taxonomic studies on the ferns in Xizang have partially uncovered the fern diversity in Medog (Wu 1983; Ching and Ling 1984a, 1984b; Zhang et al. 2004); recent work has added more new species and new records to the fern flora of this region (Wei et al. 2018; Fan et al. 2021; Qiu et al. 2022). As a continuous effort to clarify the species diversity of pteridophytes in Medog, we conducted a 12-day fieldwork in Medog in October 2021. During that trip, we collected some specimens of a species of *Hymenasplenium* that were obviously different from all other species of the genus. Our later morphological study and phylogenetic analysis confirmed that these specimens represent a distinctive species.

## ﻿Materials and methods

### ﻿Morphological studies

Morphological characters of the new species were observed in the field. Herbarium specimens of *Hymenasplenium* at KUN and PYU were studied. Digital specimens of other related species of *Hymenasplenium* were examined from the online database CVH (https://www.cvh.ac.cn/) and JSTOR Global Plants (https://plants.jstor.org/). Spore samples were taken from the type specimens and coated with gold particles using the BAL-TEC SCD 005 Cool Sputter Coater (BAL-TECAG., Liechtenstein) and imaged via a QUANTA 200 Scanning Electron Microscope (SEM; FEI Co., USA) at Yunnan University, Kunming, China.

### ﻿Phylogenetic analyses

To clarify the phylogenetic position of the new species, we sampled representatives of all the three major clades of the genus and six subclades in the Old World clade (Xu et al. 2018b). DNA sequences from five plastid markers (*atpB*, *psbA*, *rbcL*, *rps4* & *rps4-trnS*, and *trnL* & *trnL-F*) of 102 accessions representing 40 species of *Hymenasplenium* were sampled. Sixteen species of *Asplenium* were used as outgroups. Voucher information and GenBank accession numbers for each sampled taxon are provided in Appendix I.

Total genomic DNA was extracted from silica-gel-dried leaves using the TIANGEN plant genomic DNA extraction kit (TIANGEN Biotech., Beijing, China) following the manufacturers’ protocols. Five plastid markers (*atpB*, *psbA*, *rbcL*, *rps4* & *rps4-trnS*, and *trnL* & *trnL-F*) were selected for amplification and sequencing. The PCR system uses the ready-to-use rapid PCR master mix gold Mix (green) 25 μl amplification system developed by Beijing Qingke Xinye Biotechnology Co., Ltd. The new sequences were viewed and edited using Sequencher v.4.14 (Gene Codes Corporation, Ann Arbor, Michigan). The total sequences were automatically aligned in MAFFT ver. 7 (Katoh and Standley 2013) and manually adjusted in BioEdit (Hall 1999). jModeltest2 (Darriba et al. 2012) was used to select the best-fitting likelihood model for maximum likelihood (ML; Felsenstein 1973) and Bayesian analyses. The Akaike information criterion (Akaike 1974) was used to select among models instead of the hierarchical likelihood ratio test, following Pol (2004) and Posada and Buckley (2004). Maximum likelihood (ML) bootstrapping was conducted with 1000 rapid bootstrap (BS) analyses followed by a search for the best-scoring tree in a single run in RAxML v. 8 (Stamatakis et al. 2008). Bayesian inference (BI) was conducted using MrBayes 3.1.2 (Huelsenbeck and Ronquist 2001) with two runs of four Markov chain Monte Carlo chains, each beginning with a random tree and sampling one tree every 1000 generations for 10,000,000 generations. Both ML and BI analyses were conducted on Cipres (Miller et al. 2010).

## ﻿Results and discussion

### ﻿Morphological comparison

Like most species in *Hymenasplenium*, the new species have long-creeping rhizome, once-pinnate laminae, asymmetrical pinnae, reddish-brown rachis, and elliptic to reniform spores, but can be distinguished from other species in the genus by the combined characters of pinna apex obtuse to rounded, ascending pinnae, relatively fewer pairs of pinnae, and pinna-marginal teeth entire, veins terminating just below marginal teeth. The pinna shape of *H.tholiformis* is most distinct in the genus, with acroscopic margins curved and irregularly toothed, basiscopic margins truncate or slightly curved and entire, and pinna apex obtuse to rounded. A few species in the genus sometimes also have pinna apex obtuse, for example, *H.apogamum* (N.Murak. & Hatan.) Nakaike, *H.szechuanense* (Ching) Viane & S.Y. Dong (Table 1), but none of them have obviously curved margins on the acroscopic side of pinnae.

**Table 1.** Comparison of morphological characters to differentiate *Hymenaspleniumtholiformis*, *H.szechuanense*, *H.apogamum*, and *H.pseudobscurum*.

Characters	*H.tholiformis*	*H.szechuanense*	*H.apogamum*	*H.pseudobscurum*
Size of lamina	13–16 × 3–5 cm	15–25 × 3–5 cm	10–20 × 3–5 cm	20–25 × 5–10 cm
Number of lateral pinnae	15–21 pairs	20–25 pairs	15–25 pairs	15–30 pairs
Pinna shape	trapeziform to trapeziform-lunate	trapeziform	quadrangular-trapeziform	trapeziform-falcate
Size of middle pinnae	2.5–3 × 0.6–1 cm	1.5–2.3 × 0.7–1 cm	2–3.5 × 0.6–1 cm	2.5–4 × 0.8–1.8 cm
Shape of pinna apex	obtuse to rounded	truncate to obtuse	obtuse to subacute	obtuse to subacute
Stipe color	shiny, black purple	shiny, dark purple	shiny, purple	not shiny or purple
Rachis color	purple	purple	purple	grayish green
Teeth	entire	retuse	entire	entire
Sori position	medial	inframedial	medial	supramedial
Indusium	single	single	single	double
Number of basalveins lacking	3–4	3–4	1–2	3–5

### ﻿Molecular phylogenetic analyses

The alignment of five plastid markers was 5,309 bp, of which 3,848 sites were identical, 938 characters were parsimony informative, and 523 variable characters were parsimony-uninformative. A total of 12 sequences are newly generated for this study (Appendix I). The monophyly of *Hymenasplenium* was confirmed by our reconstructed phylogeny. *Hymenaspleniumtholiformis* was strongly supported as a member of the *H.excisum* subclade in the Old World clade (Xu et al. 2018b). Its affinity with *H.pseudobscurum* Viane was weakly supported (Fig. 2). To date, only three species have been recognized in the *H.excisum* subclade (Xu et al. 2018b); our discovery adds one more species to this subclade. Within the subclade, *H.tholiformis* is different from *H.obscurum* (Blume) Tagawa and *H.pseudobscurum* by having black-purple to dark purple petioles and pinna apex obtuse to rounded, and different from *H.excisum* (C. Presl) S. Lindsay by having smaller habit and truncate or slightly curved margins of the basiscopic side of pinnae.

### ﻿Taxonomic treatment

#### ﻿ Hymenasplenium tholiformis

Taxon classificationPlantaePolypodialesAspleniaceae

Liang Zhang, W.B. Ju & K.W. Xu sp. nov.

37890425-D08B-5B47-B6E6-7408B5226FBA

urn:lsid:ipni.org:names:77302869-1

##### Type.

China. Xizang: Medog County, Beibeng Xiang, Xirang, ca. 600 m from the Yarlung Zangbo River, 29°11'17.63"N, 95°03'42.27"E, 720 m elev., 28 Oct 2021, *Liang Zhang & Wen-Bin Ju 4781* (holotype: KUN1543824!; isotypes: CDBI!, KUN!).

##### Diagnosis.

*Hymenaspleniumtholiformis* is morphologically most similar to *H.szechuanense*, but different by having larger pinnae (middle pinnae 2.5–3 cm vs. 1.5–2.3 cm), extremely ascending upper pinnae (vs. spreading or slightly ascending), curved margins of acroscopic side of pinnae (vs. truncate), and pinna-marginal teeth entire and veins terminating just below marginal teeth (vs. pinna-marginal teeth retuse to emarginate and veins terminating just below these notches).

##### Description.

**Figure 1.**
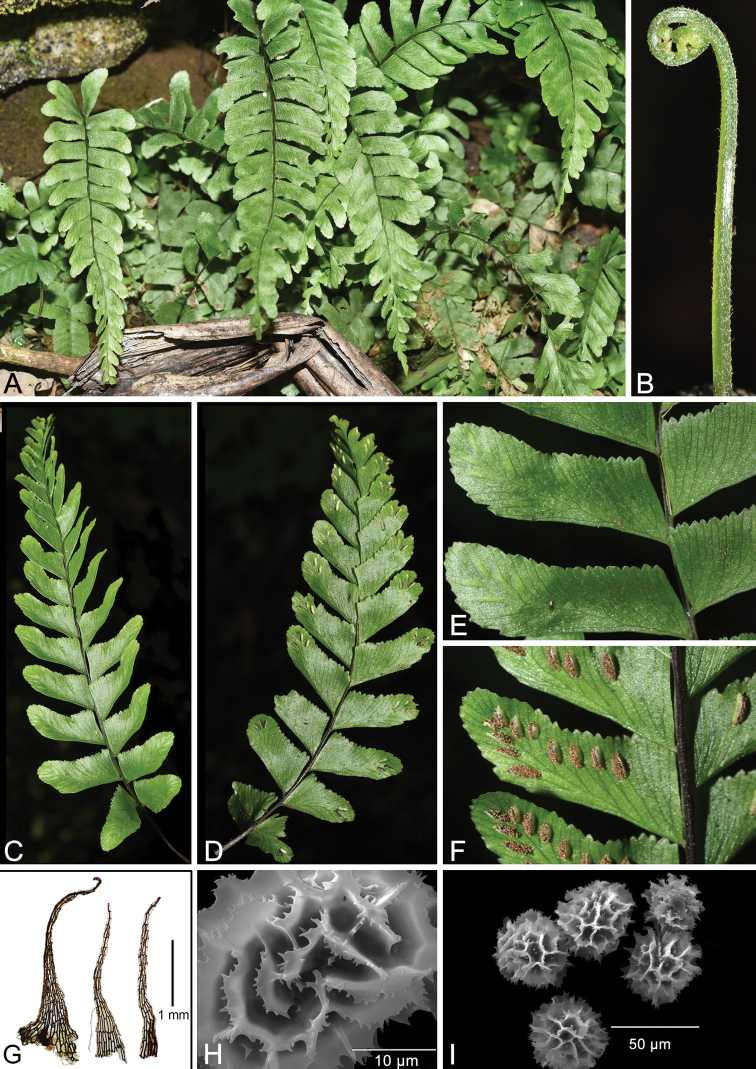
*Hymenaspleniumtholiformis* sp. nov. **A** habit **B** crozier **C** adaxial lamina **D** abaxial lamina **E** adaxial pinnae **F** abaxial pinnae, showing sori **G** rhizome scales **H** spore surface **I** spores. Photos credit: **A–F** by L. Zhang; **G–I** by W.-Z. Ma & Y.-L. Qiu.

Plants perennial, 20–36 cm. Rhizome long-creeping, ca. 2 mm in diam., apex scaly; scales dark brown, lanceolate or narrowly triangular, 0.5–1 × 0.2–0.3 mm, margins entire; roots yellowish brown when dried. Fronds remote, 10–12 mm apart, subglabrous; stipe shiny, black purple, 8–13 cm long, base ca. 2–3 mm in diam., with scales similar to those on rhizome; lamina herbaceous, once pinnate, narrowly oblong to lanceolate, 13–16 × 3–5 cm, base truncate and slightly reduced, apex acuminate to caudate; rachis 0.5–1 mm in diam., wingless, narrowly grooved adaxially, shiny, glabrous, black purple to dark purple; pinnae 15–21 pairs, trapeziform to trapeziform-lunate, basal pinnae nearly opposite, spreading or slightly ascending, upper pinnae alternate, extremely ascending, middle pinnae alternate, ascending, 2.5–3 × 0.6–1 cm, dimidiate, pinna asymmetrical, base largest, upper part of pinna enlarged, similar width as, or slightly wider than, the middle part of pinna, apex obtuse to rounded, acroscopic margins curved and irregularly toothed, teeth entire, basiscopic margins truncate or slightly curved and entire (Fig. 1). Veins visible on both sides of pinnae, free, forking and terminating in marginal teeth, 3–4 basal basiscopic veins lacking. Sori medial, linear or semi-elliptic, 5–8 pairs per pinna (Fig. 1), 2–3 mm long; indusia persistent, brown, linear or semi-elliptic, membranous, entire, opening toward costa. Spores elliptic to reniform, perispore fimbriate-alate, 43–47 μm in diam. (Fig. 1); 32 spores per sporangium.

##### Distribution and conservation assessment.

*Hymenaspleniumtholiformis* is endemic to Medog County. Currently, only one large population with ca. 35 individuals was found. According to IUCN Red List criteria B2a or D (IUCN 2022), this species should be listed as critically endangered (CR). More extensive fieldwork at low elevations in nearby mountains will be needed to accurately assess its conservation status.

**Figure 2.**
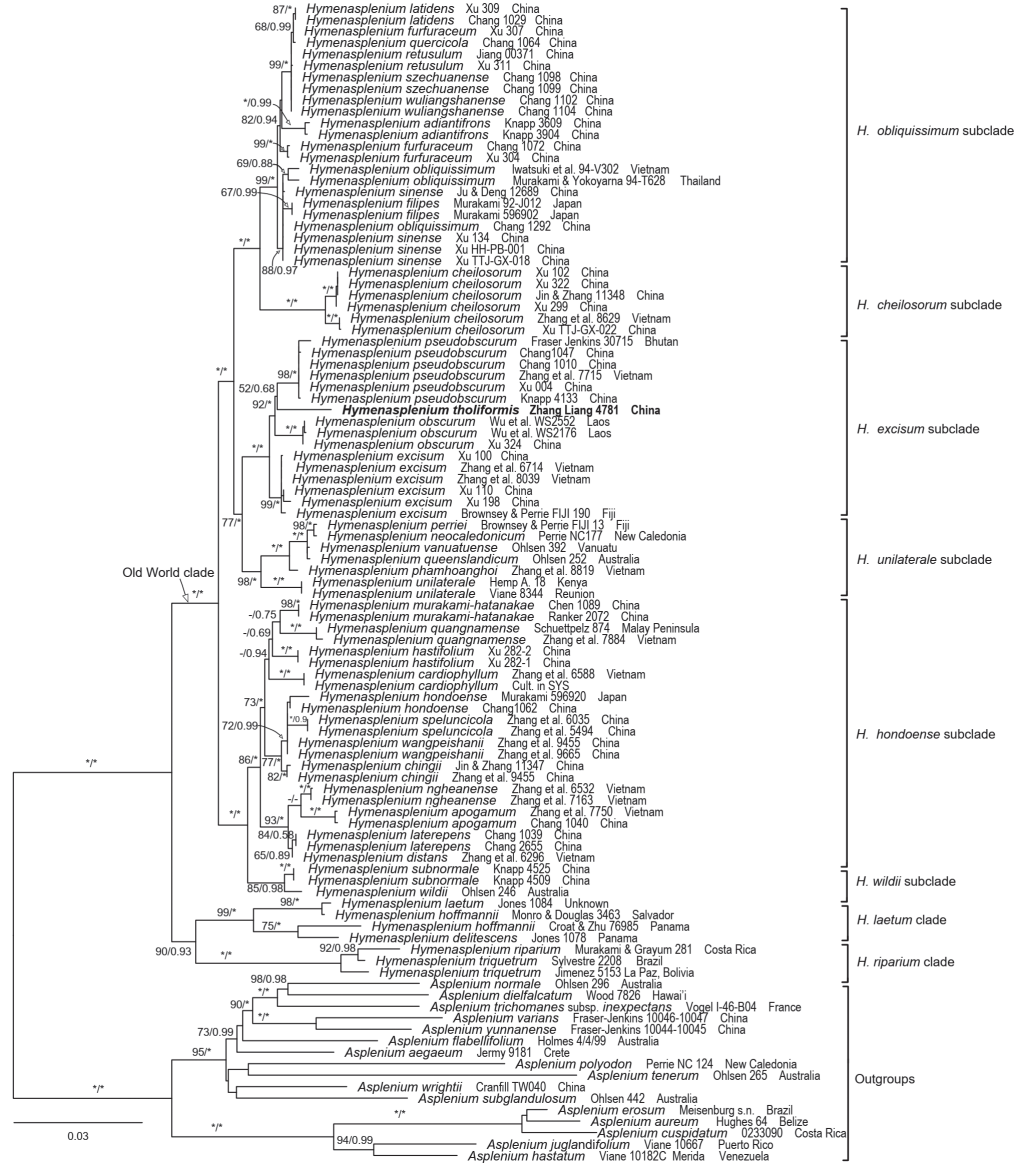
Maximum likelihood phylogeny of *Hymenasplenium* based on five plastid markers (*atpB*, *psbA*, *rbcL*, *rps4* & *rps4-trnS*, and *trnL* & *trnL-F*). The numbers associated with branches are maximum likelihood bootstrap support (MLBS) and Bayesian posterior probability (BIPP). The asterisk indicates MLBS = 100, BIPP = 1.00. The subclades are indicated following Xu et al. (2018b).

##### Ecology.

*Hymenaspleniumtholiformis* was observed in a shady place at the bottom of a large rock in the disturbed forest, at an elevation of 720 m, ca. 600 m from the Yarlung Zangbo River. High humidity and cool conditions are important for the growth of the new species.

##### Etymology.

The specific epithet alludes to dome shape of pinna apex.

##### Vernacular name.

yuan ding mo ye tie jiao jue (圆顶膜叶铁角蕨; Chinese name).

##### Comments.

In Medog County, ferns are highly diverse along the Yarlung Zangbo River and its tributaries. In this region, at elevations between 650 m and 4500 m, we have discovered four species in three subclades of *Hymenasplenium*, including *H.cheilosorum* (Kunze ex Mett.) Tagawa in the *H.cheilosorum* subclade, *H.obliquissimum* (Hayata) Sugim. in the *H.obliquissimum* subclade, and *H.excisum* and *H.tholiformis* in the *H.excisum* subclade. Of the four species, *H.tholiformis* is distributed at the lowest elevation, while *H.obliquissimum* is at the highest elevation between 2100 m to 2250 m.

## Supplementary Material

XML Treatment for Hymenasplenium tholiformis

## ﻿Appendix I

Voucher specimens and GenBank accession numbers for DNA sequences used in this study. Information is presented in the following order: species, voucher, locality, GenBank numbers for *rbcL*, *trnL* intron & *trnL-F* spacer, *rps4* & *rps4-trnS*, *atpB*, *psbA*. “*” represents the newly published sequences in this study; “–” no data. Herbaria acronyms follow Index Herbariorum (Thiers 2016).

***Aspleniumaegaeum*** Lovis, Reichst. & Greuter in Reichst.; Jermy 9181 (BM); Crete; AY300103 (Schneider et al. 2005); AY300050 (Schneider et al. 2005); AY549774 (Schneider et al. 2005); –; –. ***A.aureum*** Cav.; Hughes 64 (BM); Belize; AF240651 (Pinter et al. 2002); AF240667 (Pinter et al. 2002); AY549759 (Pinter et al. 2002); –; –. ***A.cuspidatum*** Lam.; Grantham & Parsons 0233090 (UC); Costa Rica; AY300111 (Schneider et al. 2004); AY300058 (Schneider et al. 2004); AY549760 (Schneider et al. 2004); –; –. ***A.dielfalcatum*** Viane; Wood 7826 (PTBG); Hawaii; AY549738 (Schneider et al. 2005); AY549841 (Schneider et al. 2005); AY549787 (Schneider et al. 2005); –; –. ***A.erosum*** Maxon; Unknow; –; KX397706 (Germain-Aubrey,C. C et al., Unpublished); –; –; –; –. ***A.flabellifolium*** Cav.; Holmes 4/4/99 (BM); Australia; AY300115 (Schneider et al. 2005); AY300062 (Schneider et al. 2005); AY549779 (Schneider et al. 2005); –; –. ***A.hastatum*** Klotzsch ex Kunze; Viane 10182C; Merida, Venezuela; GU929869 (Leroux et al. 2011); –; –; –; –. ***A.juglandifolium*** Lam.; Viane 10667; Puerto Rico; GU929870 (Leroux et al. 2011); –; –; –; –. ***A.normale*** D. Don; Ohlsen 296 (MELU); Queensland, Australia; KP774926 (Ohlsen et al. 2015); KP851904 (Ohlsen et al. 2015); KP835419 (Ohlsen et al. 2015); –; –. ***A.polyodon*** G. Forst; Perrie NC 124 (WELT); New Caledonia; KP774900 (Ohlsen et al. 2015); KP835397 (Ohlsen et al. 2015); KP835433 (Ohlsen et al. 2015); –; –. ***A.subglandulosum*** subsp. ***papaverifolium*** (Kunze) Salvo, Prada & Consuelo Díaz; Prada & Consuelo Díaz Ohlsen 442 (MELU); Victoria, Australia; KP774929 (Ohlsen et al. 2015); KP851909 (Ohlsen et al. 2015); KP835456 (Ohlsen et al. 2015); –; –. ***A.tenerum*** G. Forst; Ohlsen 265 (MELU); Queensland, Australia; KP774858 (Ohlsen et al. 2015); KP835346 (Ohlsen et al. 2015); KP835437 (Ohlsen et al. 2015); –; –. ***A.trichomanes*** subsp. ***inexpectans*** Lovis; Vogel I-46-B04; France; AY549743 (Schneider et al. 2005); AY549846 (Schneider et al. 2005); AY549792 (Schneider et al. 2005); –; –. ***A.varians*** Wall. ex Hook. & Grev.; Fraser-Jenkins 10046–10047 (BM); China; AY300147 (Schneider et al. 2005); AY300094 (Schneider et al. 2005); –; –; –. ***A.wrightii*** D. C. Eaton ex Hook.; Cranfill TW040 (UC); Taiwan, China; AY549730 (Schneider et al. 2005); AY549833 (Schneider et al. 2005); AY549766 (Schneider et al. 2005); –; –. ***A.yunnanense*** Franch.; Fraser-Jenkins 10044–10045 (BM); China; AY300149 (Schneider et al. 2005); AY300096 (Schneider et al. 2005); AY549803 (Schneider et al. 2005); –; –. ***Hymenaspleniumadiantifrons*** (Hayata) Viane & S. Y. Dong; Knapp 3609 (P); Taiwan, China; –; MH065559 (Xu et al. 2018); MH065322 (Xu et al. 2018); –; –. ***H.adiantifrons***; Knapp 3904 (P); Taiwan, China; –; MH065560 (Xu et al. 2018); MH065323 (Xu et al. 2018); –; –. ***H.apogamum*** N. Murak. & Hatan.; Zhang et al. 7750 (CDBI, MO, VNMN); Thua Thien-Hue, Vietnam; MH065437 (Xu et al. 2018); MH065604 (Xu et al. 2018); MH065376 (Xu et al. 2018); MH065518 (Xu et al. 2018); –. ***H.apogamum***; Chang 1040 (HITBC); Yunnan, China; MH884808 (Chang et al. 2018); –; MH884830 (Chang et al. 2018); –; MH884838 (Chang et al. 2018). ***H.cardiophyllum*** (Hance) Nakaike; Sysu 2; Cult. in Sun-yat Sen University; MH065387 (Xu et al. 2018); MH065534 (Xu et al. 2018); MH065306 (Xu et al. 2018); MH065454 (Xu et al. 2018); –. ***H.cardiophyllum***; Zhang et al. 6588 (CDBI, MO, VNMN); Lang Son, Vietnam; –; MH065584 (Xu et al. 2018); –; MH065501 (Xu et al. 2018); –. ***H.cheilosorum*** (Kunze ex Mett.) Tagawa; Xu GX022 (SYS); Guangxi, China; MH065381 (Xu et al. 2018); –; MH065343 (Xu et al. 2018); MH065448 (Xu et al. 2018); –. ***H.cheilosorum***; Xu 102 (SYS); Hainan, China; MH065385 (Xu et al. 2018); –; MH065346 (Xu et al. 2018); MH065452 (Xu et al. 2018); –. ***H.cheilosorum***; Xu 299 (SYS); Guangxi, China; MH065402 (Xu et al. 2018); –; MH065350 (Xu et al. 2018); MH065473 (Xu et al. 2018); –. ***H.cheilosorum***; Xu 322 (SYS); Yunnan, China; MH065405 (Xu et al. 2018); –; MH065352 (Xu et al. 2018); MH065476 (Xu et al. 2018); –. ***H.cheilosorum***; Zhang et al. 8629 (CDBI, MO, PHH); Lam Dong, Vietnam; MH065415 (Xu et al. 2018); –; MH065357 (Xu et al. 2018); MH065487 (Xu et al. 2018); –. ***H.cheilosorum***; Jin & Zhang 11348 (CDBI); Yunnan, China; –; MH065571 (Xu et al. 2018); MH065359 (Xu et al. 2018); MH065489 (Xu et al. 2018); –. ***H.chingii*** K. W. Xu, Li Bing Zhang & W. B. Liao; Jin & Zhang 11347 (CDBI); Yunnan, China; MH065428 (Xu et al. 2018); MH065586 (Xu et al. 2018); MH065364 (Xu et al. 2018); MH065502 (Xu et al. 2018); –. ***H.chingii***; Zhang et al. 9455 (CDBI); Guizhou, China; MH065430 (Xu et al. 2018); MH065588 (Xu et al. 2018); MH065333 (Xu et al. 2018); MH065504 (Xu et al. 2018); –. ***H.delitescens*** (Maxon) L. Regalado & Prada; Jones 1078 (MO); Panama; MH065443 (Xu et al. 2018); MH065609 (Xu et al. 2018); MH065338 (Xu et al. 2018); MH065522 (Xu et al. 2018); –. ***H.distans*** Li Bing Zhang, K. W. Xu & Liang Zhang; Zhang et al. 6296 (CDBI, MO, VNMN); Hoa Binh, Vietnam; –; MH065591 (Xu et al. 2018); MH065335 (Xu et al. 2018); MH065507 (Xu et al. 2018); –. ***H.excisum*** (C. Presl) S. Linds.; Brownsey & Perrie FIJI 190 (WELT); Fiji; KP774884 (Ohlsen et al. 2015); KP851914 (Ohlsen et al. 2015); KP851882 (Ohlsen et al. 2015); –; –. ***H.excisum***; Zhang et al. 8039 (CDBI, MO, VNMN); Quang Nam, Vietnam; MH065419 (Xu et al. 2018); MH065573 (Xu et al. 2018); MH065361 (Xu et al. 2018); –; –. ***H.excisum***; Xu 100 (SYS); Hainan, China; MH065383 (Xu et al. 2018); MH065531 (Xu et al. 2018); MH065344 (Xu et al. 2018); MH065450 (Xu et al. 2018); –. ***H.excisum***; Xu 110 (SYS); Hainan, China; MH065384 (Xu et al. 2018); MH065532 (Xu et al. 2018); MH065345 (Xu et al. 2018); MH065451 (Xu et al. 2018); –. ***H.excisum***; Xu 198 (SYS); Hainan, China; MH065394 (Xu et al. 2018); MH065541 (Xu et al. 2018); MH065341 (Xu et al. 2018); MH065462 (Xu et al. 2018); –. ***H.excisum***; Zhang et al. 6714 (CDBI, MO, VNMN); Bac Kan, Vietnam; MH065436 (Xu et al. 2018); MH065603 (Xu et al. 2018); MH065375 (Xu et al. 2018); MH065517 (Xu et al. 2018); –. ***H.filipes*** (Copel.) Sugim., Murakami 596902 (TI); Kagoshima, Japan; U30605 (Hasebe et al. 1995); –; –; –; –. ***H.filipes***; Murakami 92-J012; Kagoshima, Japan; AB016176 (Murakami et al. 1998); –; –; –; –. ***H.furfuraceum*** (Ching) Viane & S. Y. Dong; Xu 304 (SYS); Yunnan, China; MH065406 (Xu et al. 2018); MH065553 (Xu et al. 2018); MH065353 (Xu et al. 2018); MH065477 (Xu et al. 2018); –. ***H.furfuraceum***; Xu 307 (SYS); Yunnan, China; MH065409 (Xu et al. 2018); MH065557 (Xu et al. 2018); MH065320 (Xu et al. 2018); MH065481 (Xu et al. 2018); –. ***H.furfuraceum***; Chang1072 (HITBC); Yunnan, China; MW194198 (Zhang et al. 2021); –; MW194221 (Zhang et al. 2021); –; MW194182 (Zhang et al. 2021). ***H.hastifolium*** Ke Wang Xu, Li Bing Zhang &W. B. Liao; Xu 282-2 (SYS); Guangxi, China; MH065397 (Xu et al. 2018); MH065546 (Xu et al. 2018); MH065312 (Xu et al. 2018); MH065468 (Xu et al. 2018); –. ***H.hastifolium***; Xu 282-1 (SYS); Guangxi, China; MH065398 (Xu et al. 2018); MH065547 (Xu et al. 2018); MH065313 (Xu et al. 2018); MH065469 (Xu et al. 2018); –. ***H.hoffmannii*** (Hieron.) L. Regalado & Prada; Croat & Zhu 76985 (MO); Canal, Panama; MH065441 (Xu et al. 2018); MH065607 (Xu et al. 2018); MH065378 (Xu et al. 2018); –; –. ***H.hoffmannii***; Monro & Douglas 3463 (MO); Santa Ana, El Salvador; MH065442 (Xu et al. 2018); MH065608 (Xu et al. 2018); MH065337 (Xu et al. 2018); MH065521 (Xu et al. 2018); –. ***H.hondoense*** (N.Murak. & Hatan.) Nakaike; Murakami 596920 (KYO); Kouchi, Japan; AB014705 (Murakami et al. 1999); –; –; –; –. ***H.hondoense***; Chang1062 (HITBC); Yunnan, China; MH884814 (Chang et al. 2018); –; MH884833 (Chang et al. 2018); –; MH884840 (Chang et al. 2018). ***H.laetum*** (Sw.) L. Regalado & Prada; Jones 1155 (TUR); –; –; –; –; KM114105 (Lehtonen,S et al., Unpublished); –. ***H.laterepens*** N. Murak. & X. Cheng ex Y. Fen Chang & K. Hori; Chang 2655 (HITBC); Yunnan, China; MH884806 (Ohlsen et al. 2015); –; –; –; –. ***H.laterepens***; Chang 1039 (HITBC); Yunnan, China; MH884807 (Chang et al. 2018); –; MH884829 (Chang et al. 2018); –; MH884837 (Chang et al. 2018). ***H.latidens*** (Ching) Viane & S. Y. Dong; Knapp 3672 (P); Taiwan, China; –; MH065566 (Xu et al. 2018); –; –; –. ***H.latidens***; Xu 309 (SYS); Yunnan, China; MH065407 (Xu et al. 2018); MH065554 (Xu et al. 2018); MH065318 (Xu et al. 2018); MH065478 (Xu et al. 2018); –. ***H.latidens***; Y.-F. Chang1029 (HITBC); Yunnan, China; MW194204 (Zhang et al. 2021); –; MW194219 (Zhang et al. 2021); –; MW194180 (Zhang et al. 2021). ***H.murakami***-hatanakae Nakaike; Ranker 2072 (COLO); Taiwan, China; EF452140 (Schuettpelz et al. 2007); – (Schuettpelz et al. 2007); – (Schuettpelz et al. 2007); EF452020 (Schuettpelz et al. 2007); –. ***H.murakami***-***hatanakae*** Nakaike; C.-C. Chen 1089 (HITBC); Taiwan, China; MH884823 (Chang et al. 2018); –; –; –; –. ***H.neocaledonicum*** Li Bing Zhang & K. W. Xu; PerrieNC177 (WELT); New Caledonia; KP774896 (Ohlsen et al. 2015); KP851915, (Ohlsen et al. 2015); KP851878 (Ohlsen et al. 2015); –; –. ***H.ngheanense*** Li Bing Zhang, K. W. Xu & N. T. Lu; Zhang et al. 6532 (CDBI, MO, VNMN); Phu Tho, Vietnam; MH065426 (Xu et al. 2018); MH065583 (Xu et al. 2018); MH065331 (Xu et al. 2018); MH065500 (Xu et al. 2018); –. ***H.ngheanense***; Zhang et al. 7163 (CDBI, MO, VNMN); Nghe An, Vietnam; MH065439 (Xu et al. 2018); MH065605 (Xu et al. 2018); MH065336 (Xu et al. 2018); MH065519 (Xu et al. 2018); –. ***H.obliquissimum*** (Hayata) Sugim.; Murakami & J. Yokoyarna 94-T628; Chiang Mai, Thailand; AB016178 (Murakami et al. 1998); –; –; –; –. ***H.obliquissimum***; lwatsuki et al. 94-V302; Hoang Lien Son, Vietnam; AB016187 (Murakami et al. 1998); –; –; –; –. ***H.obliquissimum***; Ju & Deng 12689 (CDBI); Sichuan, China; –; MH065597 (Xu et al. 2018); MH065370 (Xu et al. 2018); MH065511 (Xu et al. 2018); –. ***H.obliquissimum***; Chang1292 (HITBC); Tibet, China; MW194212 (Zhang et al. 2021); –; MW194232 (Zhang et al. 2021); –; MW194192 (Zhang et al. 2021). ***H.obscurum*** (Blume) Tagawa; Xu 324 (SYS); Yunnan, China; MH065404 (Xu et al. 2018); MH065552 (Xu et al. 2018); MH065351 (Xu et al. 2018); MH065475 (Xu et al. 2018); –. ***H.obscurum***; Zhang et al. 7715 (CDBI, MO, VNMN); Thanh Hoa, Vietnam; MH065411 (Xu et al. 2018); MH065567 (Xu et al. 2018); MH065354 (Xu et al. 2018); MH065483 (Xu et al. 2018); –. ***H.obscurum***; Wu et al. WS2176 (MO); Xiangkhoang, Laos; ON859869 *; ON859878 *; ON859875;ON859872 *; –. ***H.obscurum***; Wu et al. WS2552 (MO); Louangphrabang, Laos; ON859870 *; ON859879 *; ON859876*;ON859873 *; –. ***H.perriei*** Li Bing Zhang & K. W. Xu; Brownsey & Perrie FIJI 13 (WELT); Fiji; KP774885 (Ohlsen et al. 2015); KP851918 (Ohlsen et al. 2015); KP851880 (Ohlsen et al. 2015); –; –. ***H.phamhoanghoi*** Li Bing Zhang, K. W. Xu & T. T. Luong; Zhang et al. 8819 (CDBI, MO, PHH); Khanh Hoa, Vietnam; MH065432 (Xu et al. 2018); MH065590 (Xu et al. 2018); MH065334 (Xu et al. 2018); MH065506 (Xu et al. 2018); –. ***H.pseudobscurum*** Viane; Knapp 4133 (P); Taiwan, China; –; MH065565 (Xu et al. 2018); –; –; –. ***H.pseudobscurum***; Xu 004 (SYS); Hong Kong, China; MH065380 (Xu et al. 2018); MH065530 (Xu et al. 2018); MH065342 (Xu et al. 2018); MH065447 (Xu et al. 2018); –. ***H.pseudobscurum***; Fraser-Jenkins30715; Bhutan; MH884826 (Chang et al. 2018); –; MH884834 (Chang et al. 2018); –; –. ***H.pseudobscurum***; Chang 1010 (HITBC); Yunnan, China; MH884827 (Chang et al. 2018); –; MH884835 (Chang et al. 2018); –; MH884841 (Chang et al. 2018). ***H.pseudobscurum***; Chang 1047 (HITBC); Yunnan, China; MH884828 (Chang et al. 2018); –; MH884836 (Chang et al. 2018); –; MH884842 (Chang et al. 2018). ***H.quangnamense*** Li Bing Zhang, K. W. Xu & Liang Zhang; Zhang et al. 7884 (CDBI, MO, VNMN); Quang Nam, Vietnam; MH065412 (Xu et al. 2018); MH065568 (Xu et al. 2018); MH065355 (Xu et al. 2018); MH065484 (Xu et al. 2018); –. ***H.quangnamense***; Schuettpelz 874; Malay Peninsula; MH884824 (Chang et al. 2018); –; –; –; –. ***H.queenslandicum*** Li Bing Zhang & K. W. Xu; Ohlsen 252 (MELU); Queensland, Australia; KP774849 (Ohlsenet al. 2015); KP851916 (Ohlsenet al. 2015); KP851879 (Ohlsen et al. 2015); –; –. ***H.quercicola*** (Ching) Viane & S. Y. Dong; Chang1064 (HITBC); Yunnan, China; MW194205 (Zhang et al. 2021); –; MW194220 (Zhang et al. 2021); –; MW194181 (Zhang et al. 2021). ***H.retusulum*** (Ching) Viane & S. Y. Dong; Jiang 00371 (SYS); Yunnan, China; MH065379 (Xu et al. 2018); MH065529 (Xu et al. 2018); MH065304 (Xu et al. 2018); MH065446 (Xu et al. 2018); –. ***H.retusulum***; Xu 311 (SYS); Yunnan, China; MH065408 (Xu et al. 2018); MH065556 (Xu et al. 2018); MH065319 (Xu et al. 2018); MH065480 (Xu et al. 2018); –. ***H.riparium*** (Liebm.) L. Regalado & Prada; Murakami & Grayum 281 (KYO); Virgen del Socorro, Costa Rica; AB014708 (Murakami et al. 1999); –; –; –; –. ***H.sinense*** K. W. Xu, Li Bing Zhang & W. B. Liao; Xu PB001 (SYS); Yunnan, China; MH065386 (Xu et al. 2018); MH065533 (Xu et al. 2018); rps4 & MH065347 (Xu et al. 2018); MH065453. (Xu et al. 2018); –. ***H.sinense***; Xu 134 (SYS); Jiangxi, China; MH065388 (Xu et al. 2018); MH065535 (Xu et al. 2018); MH065348 (Xu et al. 2018); MH065455 (Xu et al. 2018); –. ***H.sinense***; Xu GX018 (SYS); Guangxi, China; –; –; MH065349 (Xu et al. 2018); MH065465 (Xu et al. 2018); –. ***H.speluncicola*** Li Bing Zhang, K. W. Xu & H. He; Zhang et al. 5494 (CDBI, MO); Guangxi, China; –; MH065592 (Xu et al. 2018); MH065366 (Xu et al. 2018); MH065508 (Xu et al. 2018); –. ***H.speluncicola***; Zhang et al. 6035 (CDBI); Guizhou, China; –; MH065594 (Xu et al. 2018); MH065368 (Xu et al. 2018); MH065509 (Xu et al. 2018); –. ***H.subnormale*** (Copel.) Nakaike; Knapp 4525 (P); Taiwan, China; –; MH065612 (Xu et al. 2018); –; MH065526 (Xu et al. 2018); –. ***H.subnormale***; Knapp 4509 (P); Taiwan, China; –; MH065613 (Xu et al. 2018); –; MH065527 (Xu et al. 2018); –. ***H.szechuanense*** (Ching) Viane & S. Y. Dong; Chang1099 (HITBC); Sichuan, China; MW194207 (Zhang et al. 2021); –; MW194223 (Zhang et al. 2021); –; MW194183 (Zhang et al. 2021). ***H.szechuanense***; Chang1098 (HITBC); Sichuan, China; MW194206 (Zhang et al. 2021); –; MW194222 (Zhang et al. 2021); –; MW194193 (Zhang et al. 2021). ***H.tholiformis*** Liang Zhang, W.B. Ju & K.W. Xu; Zhang Liang 4781 (KUN); Tibet, China; ON859868 *; ON859877 *; –; ON859871 *; ON859874 *. ***H.triquetrum*** (N. Murak. & R. C. Moran) L. Regalado & Prada; Sylvestre 2208 (RB); Brazil; KT329398 (Mynssen et al. 2016); –; –; –; –. ***H.triquetrum***; Jimenez 5153 (MO); La Paz, Bolivia; MH065444 (Xu et al. 2018); MH065610 (Xu et al. 2018); MH065339 (Xu et al. 2018); MH065523 (Xu et al. 2018); –. ***H.unilaterale*** (Lam.) Hayata; Viane 8344; Reunion; GU929873 (Leroux et al. 2011); –; –; –; –. ***H.unilaterale***; Hemp A. 18 (BM); Kenya; AF240652 (Pinter et al. 2002); AF525232 (Pinter et al. 2002); –; –; –. ***H.vanuatuense*** Li Bing Zhang & K. W. Xu; Ohlsen 392 (MELU); Tanna, Vanuatu; KP774898 (Ohlsen et al. 2015); KP851917 (Ohlsen et al. 2015); KP851881 (Ohlsen et al. 2015); –; –. ***H.wangpeishanii*** Li Bing Zhang & K. W. Xu; Zhang et al. 9665 (CDBI); Guizhou, China; MH065423 (Xu et al. 2018); MH065577 (Xu et al. 2018); –; MH065494 (Xu et al. 2018); –. ***H.wangpeishanii***; Zhang et al. 9455 (CDBI); Guizhou, China; MH065430 (Xu et al. 2018); MH065588 (Xu et al. 2018); MH065333 (Xu et al. 2018); MH065504 (Xu et al. 2018); –. ***H.wildii*** (Bail.) D.J.Ohlsen; Ohlsen 246 (MELU); Queensland, Australia; KP774927 (Ohlsen et al. 2015); KP851919 (Ohlsen et al. 2015); KP851877 (Ohlsen et al. 2015); –; –. ***H.wuliangshanense*** (Ching) Viane & S. Y. Dong; Y.-F. Chang1102 (HITBC); Yunnan, China; MW194208 (Zhang et al. 2021); –; MW194224 (Zhang et al. 2021); –; MW194184 (Zhang et al. 2021). ***H.wuliangshanense***; Chang1104 (HITBC); Yunnan, China; MW194209 (Zhang et al. 2021); –; MW194225 (Zhang et al. 2021); –; MW194185 (Zhang et al. 2021).
